# A hidden pathophysiology of endolymphatic hydrops: case report of a patient with spontaneous intracranial hypotension presenting with sudden sensorineural hearing loss with vertigo

**DOI:** 10.3389/fneur.2024.1394534

**Published:** 2024-04-05

**Authors:** Jong Kwan Kim, Ho Yun Lee, Ha Youn Kim, Min Young Kwak

**Affiliations:** ^1^Department of Otorhinolaryngology-Head and Neck Surgery, Daejeon Eulji Medical Center, Eulji University, Daejeon, Republic of Korea; ^2^Department of Otolaryngology-Head and Neck Surgery, College of Medicine, Ewha Womans University, Seoul, Republic of Korea; ^3^Department of Radiology, Daejeon Eulji Medical Center, Eulji University, Daejeon, Republic of Korea; ^4^Department of Otorhinolaryngology-Head and Neck Surgery, Hallym University Dongtan Sacred Heart Hospital, Hallym University College of Medicine, Hwaseong, Republic of Korea

**Keywords:** intracranial hypotension, endolymphatic hydrops, MRI, hearing loss, vertigo

## Abstract

Spontaneous intracranial hypotension (SIH) is characterized by decreased cerebrospinal fluid (CSF) volume due to leakage through the dural membrane. We present the case of a patient with SIH manifested by fluctuating low-frequency hearing loss, tinnitus, and vertigo. In this patient, endolymphatic hydrops in the cochlea and saccule were visualized by means of a special sequence of inner ear magnetic resonance imaging scans, with a gadolinium-based contrast agent administered intravenously. Endolymphatic hydrops is a potential underlying pathophysiology of SIH-associated hearing impairment. We hypothesize that SIH may be a rare cause of endolymphatic hydrops.

## 1 Introduction

Spontaneous intracranial hypotension (SIH) is clinically characterized by postural headaches resulting from a cerebrospinal fluid (CSF) pressure of 6 cmH_2_0 or less in the lateral decubitus position (1). The primary cause of SIH is CSF leakage through the dural membrane resulting from a structural weakness in the dura, absence of dura around the nerve root sheaths, a congenital connective tissue defect causing structural abnormalities, osteophyte protrusions, or herniation of the spinal disc (2). The extent of CSF leakage can range from a small release, e.g., during a Valsalva maneuver, to a substantial spontaneous outflow (3).

When the volume of CSF decreases due to leakage, the brain can sag within the cranial cavity, and venodilation may occur to compensate for the fluid depletion (4). The brain descent exerts traction on the sensory nerves of the meninges and bridging veins, giving rise to headaches. The diagnosis of SIH is complex and challenging, since headaches are a major presenting symptom for a wide range of pathologies, specific diagnostic markers are lacking, and tests for SIH are invasive (5).

Herein, we present the case of a patient with SIH characterized by sudden low-frequency sensorineural hearing loss and vertigo. We demonstrate the presence of endolymphatic hydrops in this patient using delayed hybrid of reversed image of positive endolymph signal and native image of positive perilymph signal (HYDROPS) magnetic resonance imaging (MRI).

## 2 Case presentation

A 42-year-old male initially presented with tinnitus and ear fullness in both ears that developed 2 days prior. He had no significant past medical or surgical history except for hypertension, sleep apnea, and a history of frequent headaches over several years, which had been well-controlled with pain relievers. He had no dizziness or other neurologic symptoms. There was no history of trauma to the head or neck. During the month preceding admission, he had been admitted to the Department of Rehabilitation Medicine for neck pain radiating to his right arm and received physical therapy and medication.

**Figure 1:**
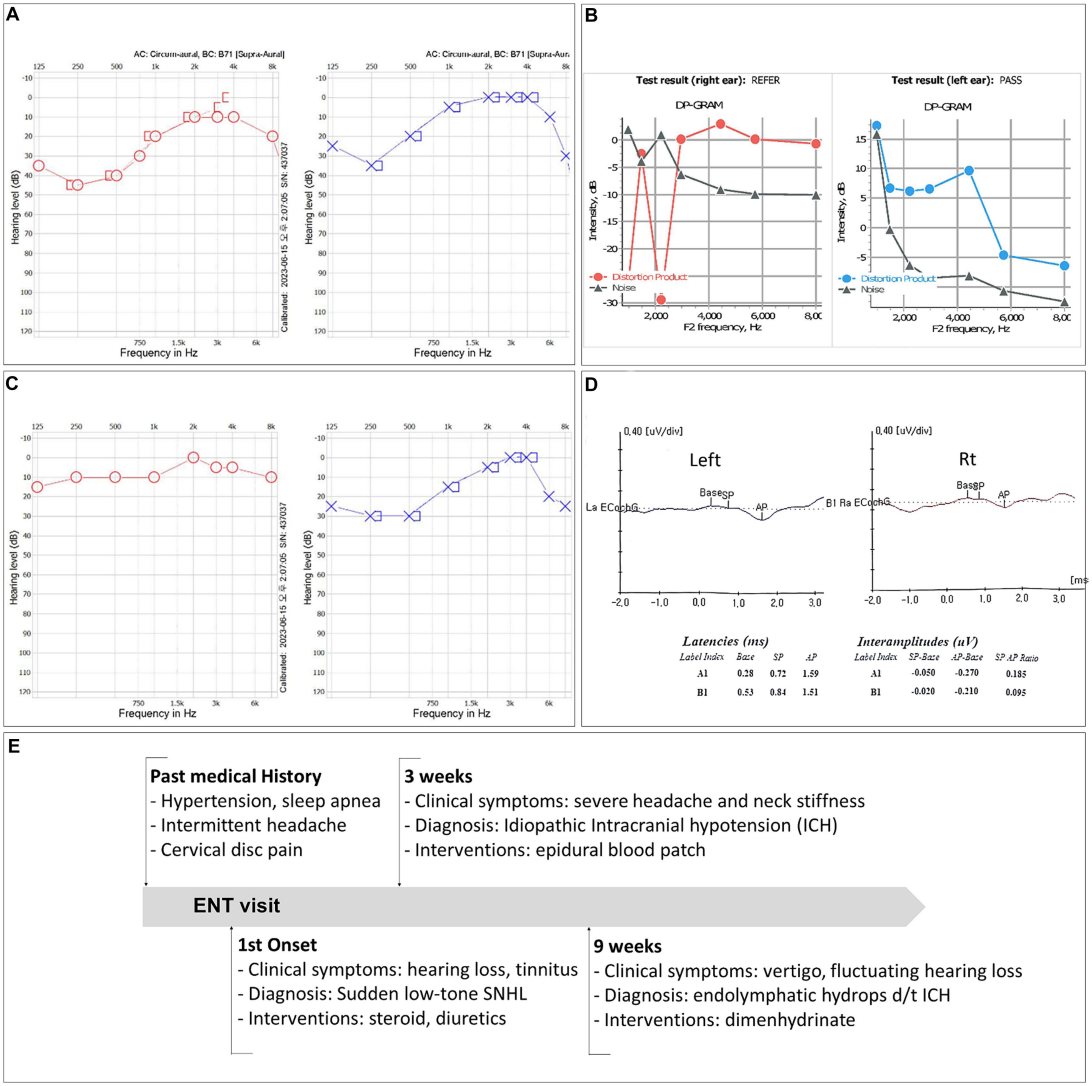
**(A)** Pure tone audiometry (PTA) test results showing low-frequency sensorineural hearing loss in both ears. **(B)** Results of a PTA test performed 2 weeks after the initiation of steroid therapy. **(C)** Distortion product otoacoustic emissions were not detected at low frequencies. **(D)** The patient’s electrocochleography test results. **(E)** Timeline of the case.

Pure tone audiometry testing indicated a moderate decline in low-frequency thresholds, with a maximum hearing loss of 45 dB (250 Hz) and 35 dB (250 Hz) in the left and right ear, respectively (Figure 1A). Distortion product otoacoustic emissions were absent at low frequencies (Figure 1C). The tinnitus, which had a minimum masking level of 41 dB, was described as a permanent and non-pulsatile sound perception in the low-frequency range (0.125 kHz).

The patient was diagnosed with idiopathic sudden sensorineural hearing loss and was treated with high-dose steroid and isosorbide solution. Two weeks after the onset of symptoms, his hearing had improved to a hearing loss of 10 dB (250 Hz) and 30 dB (250 Hz) in the right and left ear, respectively (Figure 1B). However, due to unresolved tinnitus and fluctuating ear fullness, the patient received additional intratympanic steroid injections.

After the onset of hearing loss and over the next few weeks, he developed intense headaches that were unresponsive to pain relievers, prompting a visit to the neurological emergency department of our hospital. The patient’s blood pressure was 155/92 mmHg, pulse was 94 beats/min, temperature was 36.9°C, respiratory rate was 18 breaths/min, and oxygen saturation level was 100%. The results of neurological examinations of mental status, cranial nerves, motor strength, sensation, reflexes, and cerebellar function were all completely normal.

**Figure 2:**
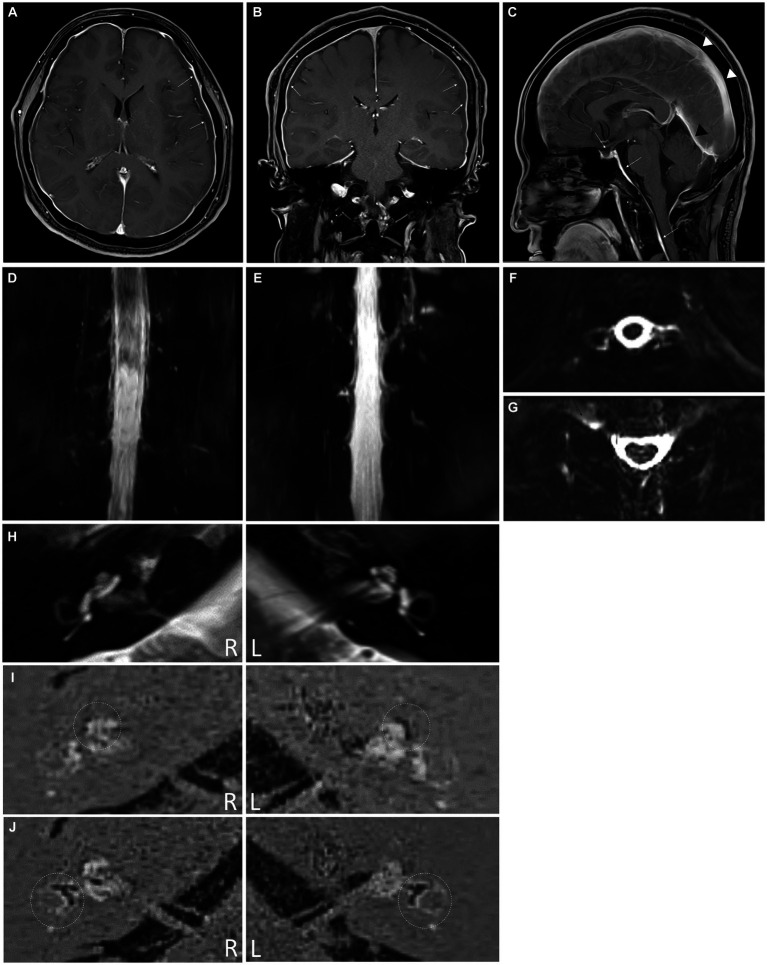
**(A)** Axial post-contrast medium T_1_-weighted magnetic resonance (MR) image showing diffuse smooth thickening of the cerebral convexity dura (white arrows) and bilateral subdural liquid collection (yellow arrows). **(B)** Coronal T_1_-weighted MR image with contrast demonstrating diffuse dural enhancement (white arrows) with bilateral subdural liquid collection (yellow arrows). **(C)** Sagittal post-contrast medium T_1_-weighted MR image showing dural enhancement of the pituitary (yellow arrow). The presence of diffuse smooth dural thickening (white arrows), which extends into the spine, indicates spontaneous intracranial hypotension, demonstrating prominent sagittal sinus (arrowheads) and transverse sinus (black arrowhead). **(D–G)** Intrathecal Gd-magnetic resonance myelography images revealing cerebrospinal fluid leakage at multiple levels (C4-5, C5-6, C6-7, and C7-T1 on right side, C4-5 on left side, and T1-2 on both sides) in the coronal **(D)** and axial views **(E)**. **(F,G)** Cerebrospinal fluid leakage is also observed at the T10-11 and T11-12 levels on both sides. **(H)** Axial T_2_-weighted magnetic resonance (MR) image showing a normal internal auditory canal in both ears. The HYDROPS MR image was generated by subtracting the positive perilymph signal from the positive perilymph image. The dotted circles indicate the cochlea **(I)** and vestibular apparatus **(J)**. The endolymphatic hydrops is detectable as a black area inside the perilymphatic space filled with the gadodiamide.

An MRI scan of the brain revealed subdural fluid collection along the bilateral convexity and thickened dural enhancement around the pituitary gland, which were evidence of a small subdural hemorrhage and intracranial hypotension (Figures 2A–C). MR myelography of the entire spine showed significant leakage at multiple levels of the retrospinal region (right C4-5, C5-6, and C6-7 and bilateral T1-2, T10-11, and T11-12) (Figures 2D–G). The patient was treated with intravenous fluids and absolute bed rest, followed by an epidural blood patch (EPB) administered by a C-arm fluoroscopically-guided injection of 10 mL of autologous blood into the C7-T1 epidural space.

After cervical EBP administration, his headaches resolved, and the patient was discharged the following day. Over the next few months, he reported intermittent mild headaches and neck stiffness with fluctuating ear fullness and tinnitus. Nine weeks after discharge, the patient returned with an acute onset of whirling-type vertigo and nausea. Videonystagmography recorded a maximum slow phase velocity nystagmus at 1.6°/s toward the left side after a head-shaking test. Electrocochleography tests (Figure 1D) and brain and spine MRI scans were performed to look for endolymphatic hydrops. The MRI scans were conducted using a 3-Tesla MRI scanner equipped with a 32-channel array head coil. To enhance the contrast for visualizing the inner ear, and since the gadolinium-based contrast agent was given as a single intravenous dose (0.2 mL/kg body weight, 4 h before MRI), a HYDROPS MR image was obtained by subtracting the T_2_-weighted 3D inversion recovery image (positive endolymphatic image) from the 3D fluid-attenuated inversion recovery image (positive perilymphatic image), thus improving the interface between the three spaces in a single image (Figures 2H–J).

The summating potential/action potential ratios were 0.095 in the right ear and 0.185 in the left ear, and the HYDROPS MR image showed significant endolymphatic hydrops in the cochlea (Figure 2I) and vestibules (Figure 2J) of both ears. The patient’s symptoms resolved shortly after taking dimenhydrinate. The timeline of the episodes and interventions for this case is presented in Figure 1E.

## 3 Discussion

Herein, we present the case of a patient exhibiting concurrent symptoms and diagnostic indicators of SIH and endolymphatic hydrops, supported by objective brain and spine MRI, inner ear HYDROPS MRI, and audiogram findings. Although several case reports of SIH with symptoms of hearing loss have been reported (6–8), it is very rare to objectively visualize endolymphatic hydrops as the pathophysiologic basis of cochlear vestibular symptoms in patients with SIH. To the best of our knowledge, there is only one other documented SIH case report, involving a 41-year-old female, where MRI examinations confirmed the presence of endolymphatic hydrops (9). However, the authors were cautious in associating the SIH with CSF leakage because leakage was not identified on the MR myelogram. Our present case report could contribute to establishing a more conclusive association between intracranial hypotension with CSF leakage and endolymphatic hydrops (9).

SIH is a highly underdiagnosed and misdiagnosed disease (10). Because of our patient’s previous history of migraines and cervical disc herniation and the fact that postural headaches and neck stiffness can be nonspecific and may overlap with other more common conditions (11), his SIH was not diagnosed. According to a recent systematic review of SIH, the condition has widely diverse signs and symptoms, and orthostatic headache, once considered an essential characteristic, is not always present (11): 20% of SIH cases had tinnitus, 28% had hearing disturbances, and 27% presented with dizziness. The authors further suggested that a significant minority of patients with SIH may have non-orthostatic headaches, normal lumbar punctures, or normal imaging results, and the diagnosis of SIH cannot be excluded in patients who do not present with all the typical features of this disorder.

A possible relationship between endolymphatic hydrops and reduced CSF pressure was first suggested by Carlborg and Farmer in 1983 (12). Although the physiopathology of this association remains unknown, it is thought that decreased CSF pressure can be transmitted through a patent cochlear aqueduct: A drop in CSF pressure causes a simultaneous, rapid decrease in perilymph pressure, leading to compensatory expansion of the endolymphatic compartment, which may clinically resemble that found in Meniere’s disease (13–15). Endolymphatic hydrops can result from a change in intracranial CSF pressure, as well as abnormal endolymph production and absorption. Cochlear nerve stretching due to the brain sagging under reduced intracranial pressure could also explain the audiovestibular symptoms of SIH (16).

Not all cases of endolymphatic hydrops result from SIH, and not every patient with SIH develops endolymphatic hydrops. However, this case illustrates that decreased CSF pressure can be a pathophysiological cause of endolymphatic hydrops and that SIH can manifest vestibulocochlear symptoms through an endolymphatic hydrops-mediated mechanism.

Remarkably, treatment approaches for endolymphatic hydrops and SIH differ greatly. The established treatment for SIH is conservative management: strict bed rest, hydration, controlled caffeine intake, avoidance of upright sitting, and EBP to tamponade the CSF leak. A previous study proposed that caffeine can increase CSF secretion (17), whereas a preclinical study conducted in 2023 indicated that caffeine administration could decrease CSF secretion (18). Additionally, some reports suggest that caffeine exerts a vasoconstrictive effect, mitigating venous engorgement and dilation by reducing intracranial CSF volume in patients with SIH (4, 19). In contrast, the therapeutic approach for endolymphatic hydrops involves dietary modifications, such as salt and caffeine restriction. Because of its vasoconstrictive property, caffeine may lead to a reduced blood supply to the inner ear. Diuretics, commonly employed in endolymphatic hydrops treatment, can also contribute to a lowering of the intracranial pressure. One intriguing case report details an initial misdiagnosis of SIH as endolymphatic hydrops, where the subsequent treatment with acetazolamide and betahistine exacerbated headache and nausea symptoms (13).

Therefore, as with our patient, individuals experiencing acute hearing loss and dizziness should not have their previous histories (including headache, migraine, or neck stiffness) underestimated. If present, intracranial hypotension should be cautiously considered as a potential differential diagnosis. A comprehensive assessment of the medical history (including headache, neck stiffness, trauma, or procedures such as lumbar puncture), neurological tests, and radiologic examinations may be valuable in determining the mechanisms underlying idiopathic sudden sensorineural hearing loss with dizziness.

## Data availability statement

The original contributions presented in the study are included in the article/supplementary material, further inquiries can be directed to the corresponding author.

## Ethics statement

The studies involving humans were approved by Institutional Review Boards of the Eulji University Daejeon Eulji Medical Center. The studies were conducted in accordance with the local legislation and institutional requirements. Written informed consent for participation was not required from the participants or the participants’ legal guardians/next of kin in accordance with the national legislation and institutional requirements. Written informed consent was obtained from the individual(s), and minor(s)’ legal guardian/next of kin, for the publication of any potentially identifiable images or data included in this article.

## Author contributions

JK: Writing – original draft, Writing – review & editing, Conceptualization, Methodology, Resources. HL: Conceptualization, Supervision, Validation. HK: Supervision, Writing – original draft, Conceptualization, Validation, Writing – review & editing. MK: Writing – original draft, Writing – review & editing.
